# Predicting the Activity Coefficients of Free-Solvent for Concentrated Globular Protein Solutions Using Independently Determined Physical Parameters

**DOI:** 10.1371/journal.pone.0081933

**Published:** 2013-12-04

**Authors:** Devin W. McBride, Victor G. J. Rodgers

**Affiliations:** B2K Group (Biotransport & Bioreaction Kinetics Group), Center for Bioengineering Research, Department of Bioengineering, University of California Riverside, Riverside, California, United States of America; Jacobs University Bremen, Germany

## Abstract

The activity coefficient is largely considered an empirical parameter that was traditionally introduced to correct the non-ideality observed in thermodynamic systems such as osmotic pressure. Here, the activity coefficient of free-solvent is related to physically realistic parameters and a mathematical expression is developed to directly predict the activity coefficients of free-solvent, for aqueous protein solutions up to near-saturation concentrations. The model is based on the free-solvent model, which has previously been shown to provide excellent prediction of the osmotic pressure of concentrated and crowded globular proteins in aqueous solutions up to near-saturation concentrations. Thus, this model uses only the independently determined, physically realizable quantities: mole fraction, solvent accessible surface area, and ion binding, in its prediction. Predictions are presented for the activity coefficients of free-solvent for near-saturated protein solutions containing either bovine serum albumin or hemoglobin. As a verification step, the predictability of the model for the activity coefficient of sucrose solutions was evaluated. The predicted activity coefficients of free-solvent are compared to the calculated activity coefficients of free-solvent based on osmotic pressure data. It is observed that the predicted activity coefficients are increasingly dependent on the solute-solvent parameters as the protein concentration increases to near-saturation concentrations.

## Introduction

Many cells contain macromolecular crowded protein environments (mixed proteins with total concentrations between 50 – 400 g/L), and therefore, the crowded environment is an essential component of cells [1], [2]. One feature of macromolecular crowding is the deviation of the osmotic pressure from ideality called crowded protein osmotic pressure. The significance of the osmotic pressure due to these crowded proteins is that it may play a critical factor in intracellular flux as well as impact the reactive environment.

Although crowded protein environments are abundant and naturally occurring, many studies focus on single protein solutions for studying and understanding the effect(s) of crowded environments. These concentrated solutions, in which a single macromolecule is examined at high concentrations, are more convenient than crowded solutions; they can yield information about the effects of excluded volume (volume which is occupied by the macromolecule) on various phenomenon, such as reaction kinetics and thermodynamics [3].

Generally, to correct for the deviations from ideal models in crowded environments, an activity coefficient is introduced which accounts for the various interactions responsible for observations. Until now, there has been no rigorous assessment of how the activity coefficient of free-solvent is related to the solute and solution properties. Recently, the free-solvent model, introduced by van Laar [4] and developed by Yousef *et al.* [5]–[9] as be shown to give excellent predictability of the osmotic pressure of single and binary protein solutions up to near saturation. Once more, the model developed by Yousef *et al.* [5], [6] used only physically realistic and independently determinable parameters in making these excellent predictions. In this work, the free-solvent model is used to directly couple the activity coefficient of free-solvent to these parameters, thus providing, for the first time, a fundamental basis for the concentration dependency of the solution activity coefficients.

### Definition of the Activity Coefficient

Historically, the activity coefficient model for relating concentrations to chemical potential was developed to correct for non-idealities observed in many equilibrium systems. Recall, that the chemical potential for species 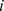 can be related to the species relative activity, 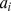, as,



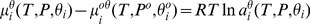
(1).

For an ideal system (with no attractive interactions), the relative activity is proportional to a composition variable, 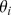 (such as 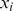, 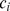, 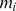, 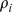, etc.). For observed non-ideal behavior, an activity coefficient, 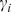, is introduced to ‘correct’ for the deviation



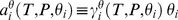
(2).

### Relationship of the Activity Coefficient to Osmotic Pressure

When a two-chamber osmometer, containing diffusible species on one side and diffusible and non-diffusible species on the other side, is separated by a semi-permeable membrane, an osmotic pressure develops which directly corresponds to the chemical potential of the diffusible species across the membrane.

Denoting the chamber containing proteins denoted as compartment II, and the chamber containing only solvent and diffusible ions denoted as compartment I, the chemical potential of species 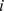 in Chamber II at pressure 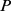, 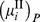, and the chemical potential of species 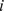 in Chamber I at pressure 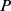, 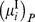, are related such that



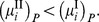



At equilibrium, assuming that the chemical potential of species 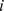 in Chamber I remains unchanged, that the temperature and pressure are constant, and that the number of diffusible species crossing the membrane is constant, then



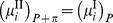



where 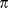, the osmotic pressure, is the increase in the pressure required to satisfy chemical potential equivalence of species 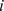 in the two chambers.

Letting



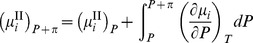



and the specific volume, 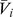, be defined as



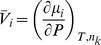



yields



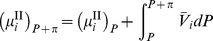



Therefore it follows that



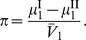



Finally, the osmotic pressure, in terms of the solvent activity, is



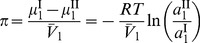
(3).

In an ideal system, the activity coefficient is unity, thus the activity is linearly related to the composition variable, and therefore the osmotic pressure is expressed as



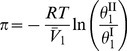
(4).

However, for a non-ideal system, such as observed in a concentrated or crowded protein environment, using the mole fraction of the solvent, 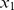, as the composition variable, the osmotic pressure is related to mole fraction as



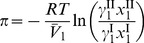
(5).

Assuming that the activity coefficient of free-solvent in compartment I (non-protein solution) is unity, 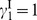, Eqn. 5 can be used to fit the experimental osmotic pressure data to determine the values of the activity coefficient of free-solvent at each protein concentration. In this work, we will reexamine the activity coefficient of free-solvent in terms of the free-solvent parameters.

### Free-Solvent Model

As early as 1916, Frazer and Myrick [10] analyzed the non-idealities in concentrated, aqueous solutions of sucrose using a free-solvent model understanding that the mole fraction of water is affected by the hydration of sucrose. When the water that interacts with sucrose was removed from the total water available in the system, the free-solvent model provided an excellent prediction of the osmotic pressure data.

More recently, the free-solvent model was revised for aqueous protein solutions in which ion binding occurs, in addition to hydration [9]. Essentially, the free-solvent model treats the protein with all associated water and salt ions as a unique species, the hydrated macromolecule. In effect, this approach renders the solution ideal with respect to the remaining, diffusible solvent species that have no attractive interactions. The modified mole fraction of the free water, 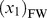, considers the hydrated macromolecule as the impermeable solute. The free-solvent model with the mole fraction of the free water, 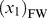, as the composition variable is



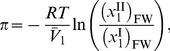
(6)


where the mole fraction of free water is the remaining moles of solvent that are not bound to the protein. Assuming the solutions is made up of *n* distinct species and *p* proteins, and letting species 1 be the solvent, species 2 through 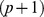 be the proteins, and species 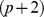 through *n* be the remaining diffusible species, the initial total moles of the solution in compartment II is 
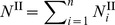
, where 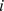 denotes each species. The final total moles of free-solvent in chamber II, after protein-solvent interactions, is 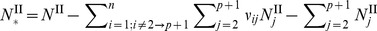, where 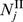 denotes the moles of protein 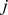 in solution and 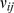 is the number of moles of species 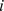 interacting with protein 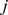 to make the hydrated protein. Then, the mole fraction of free-solvent in chamber II is
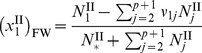
(7)


while in chamber I, the mole fraction of free-solvent is



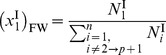
(8).

For a single protein species in a monovalent salt aqueous solution, the free-solvent model reduces to



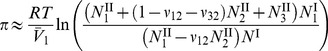
(9).

### Robustness of the Physical Parameters in the Free-Solvent Osmotic Pressure Model

The parameters of the free-solvent model have been shown to be remarkably robust and well-within independently determined values when regressed relative to measured osmotic pressure for highly concentrated protein solutions [5]–[9], [11]. As an example, the regressed hydration number, 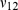, for all globular proteins measured was found to be well within the ^17^O NMR approximation of 1 g H_2_O/g globular protein [12] but more precisely determines the value to be a monolayer of water with 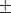
0.6% when compared to the solvent accessible surface area (SASA) of each protein. Thus, the free-solvent model is likely to provide an excellent prediction of the activity coefficient of free-solvent that is developed from only independently determined physical parameters.

### Coupling the Activity Coefficient of Free-Solvent to the Free-Solvent Model

Using the free-solvent model (Eqn. 6), the activity coefficient of free-solvent can be determined based on the ratio of the mole fractions of total water, 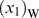, and the ratio of the mole fractions of free water, 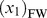. Using 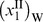 and 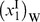 as the mole fractions of total water, and 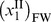 and 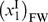 as the mole fractions of free water, setting Eqns. 5 (with 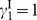) and 6 equal yields
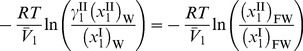
(10)


where 
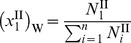
 and 
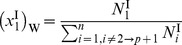
. Substituting Eqns. 7 and 8 into Eqn. 10 and solving for the activity coefficient of free-solvent, results in



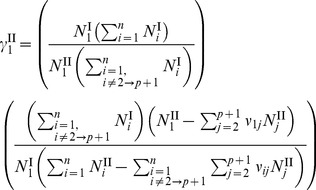
(11).

For a single protein in an aqueous solution with a single monovalent salt, the activity coefficient of free-solvent becomes



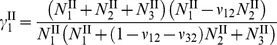
(12).

Eqn. 12 gives the relationship of the activity coefficient of free-solvent to the protein-solvent interactions and moles of species in solution. Note that, again, only measurable physical properties are necessary to determine the activity coefficient and there are no arbitrary parameters.

## Materials and Methods

The activity coefficients of free-solvent were predicted based on protein-solvent interactions (Eqn. 12) and compared to the activity coefficients of free-solvent calculated using osmotic pressure data (Eqn. 5) for two proteins: bovine serum albumin (BSA) in 0.15 M NaCl, 25°C at pH 4.5, 5.4, and 7.4 and sheep hemoglobin (Hb) in 0.1 M KCl, 0°C, pH 7.43. The model is also used to predict the activity coefficients of free-solvent for sucrose in water at 30°C.

The calculated (osmotic pressure-based) activity coefficients of free-solvent were computed at each protein concentration by solving Eqn. 5, with 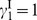, using the osmotic pressure data by Vilker *et al.* [13] for the concentrated BSA solutions (0.15 M NaCl at 25°C, pH 4.5, 5.4, and 7.4), to the osmotic pressure data by Adair, published by Dick [14], for concentrated Hb in 0.1 M KCl, 0°C, pH 7.43, and to the osmotic pressure by Frazer and Myrick [10] for concentrated sucrose solutions.

### The Activity Coefficients of Free-Solvent Based on Independently Measurable Parameters

Using the model developed for the activity coefficient of free-solvent based on protein-solvent interactions (Eqn. 12), the activity coefficients of free-solvent were predicted, for each macromolecule, using available literature values for the hydrations and ion bindings.

Since the value of hydration can vary depending on the experimental method used, here the solvent accessible surface area (SASA) was used to determine the value of hydration [9]. The SASA, computed using five molecular modeling software as previously described to compute hydration [11], was used to determine the value of hydration assuming 15.2 molecules per nm^2^ of surface area [9]. The five molecular modeling software used are Swiss-Pdb Viewer [15], MOLMOL [16], UCSF Chimera [17], VegaZZ [18], and GETAREA [19]. For Swiss-Pdb Viewer and MOLMOL a quality and precision of 6 were used, respectively, for calculating the SASA.

For BSA, three molecular structures are available (two in the Protein Data Bank (PDB: 3V03 [20] and 4F5S [21]) and a homology model (based on human serum albumin (PDB: 1BM0 [22]) [23]). Here, the hydration values used are determined from the SASA using the molecular structure obtained from homology modeling. The ion binding values of BSA were those based on the two-site model by Scatchard *et al*. [6], [24].

Similarly, for Hb the hydration value used was that of the hydration computed from the SASA of the molecular structure (PDB: 2QU0 [25]) and the ion binding value was determined by De Rosa *et al*. [26].

For sucrose, the hydration values used are those of Frazer and Myrick [10], Scatchard [27], and Einstein [28].

## Results

**Figure 1:**
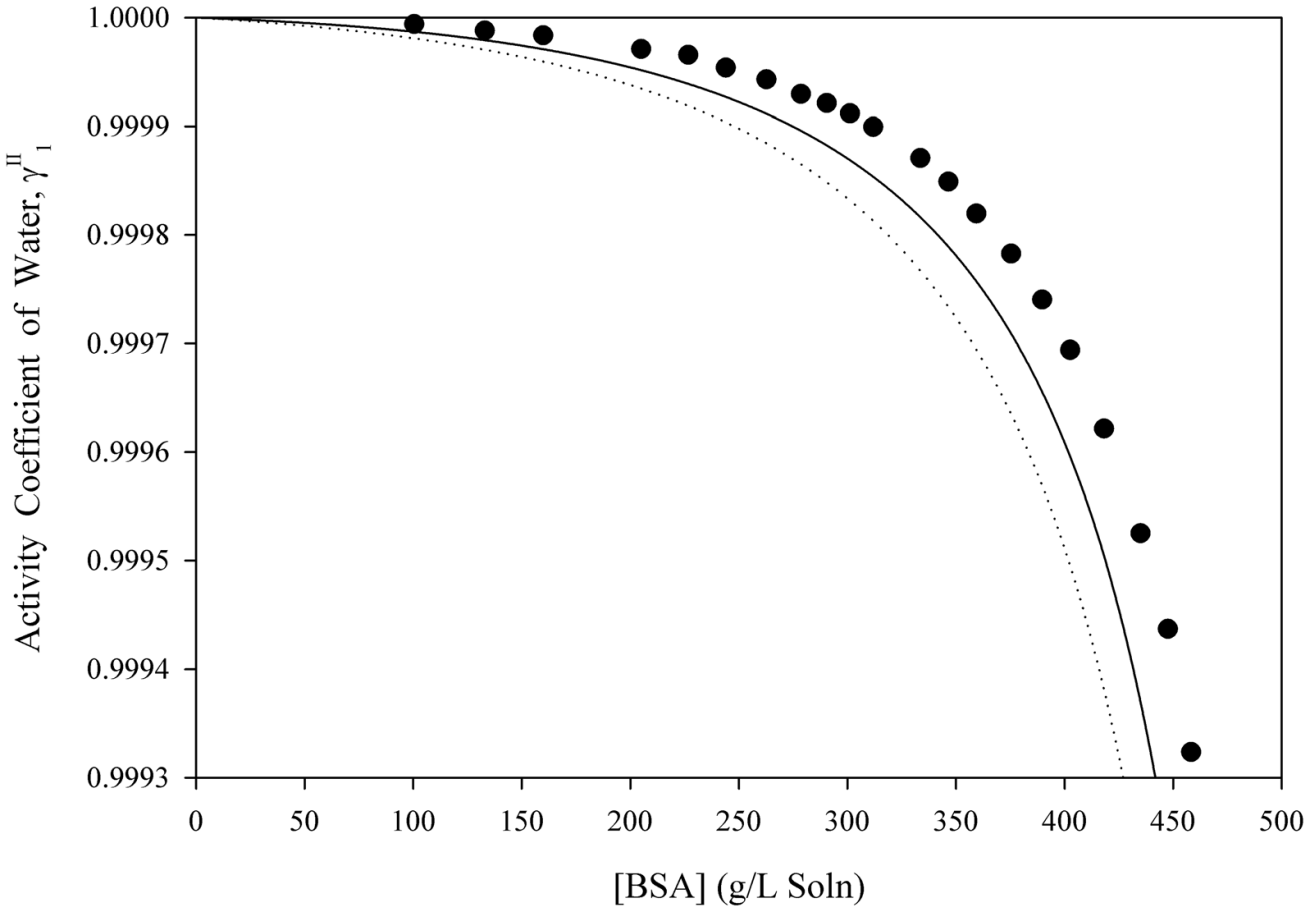
Activity Coefficient of Free-Solvent Using Literature Values of the Physical Parameters vs. Concentration for BSA in 0.15 M NaCl, pH 4.5. The calculated activity coefficients of BSA in 0.15(closed circles) are shown. The predicted activity coefficients (Eqn. 12) are plotted using the physical parameters available in literature for BSA in 0.15 M NaCl, pH 4.5 (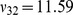 mol NaCl/mol BSA [6]): 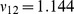 g H_2_O/g BSA (solid curve) and 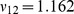 g H_2_O/g BSA (dotted curve).

**Figure 2:**
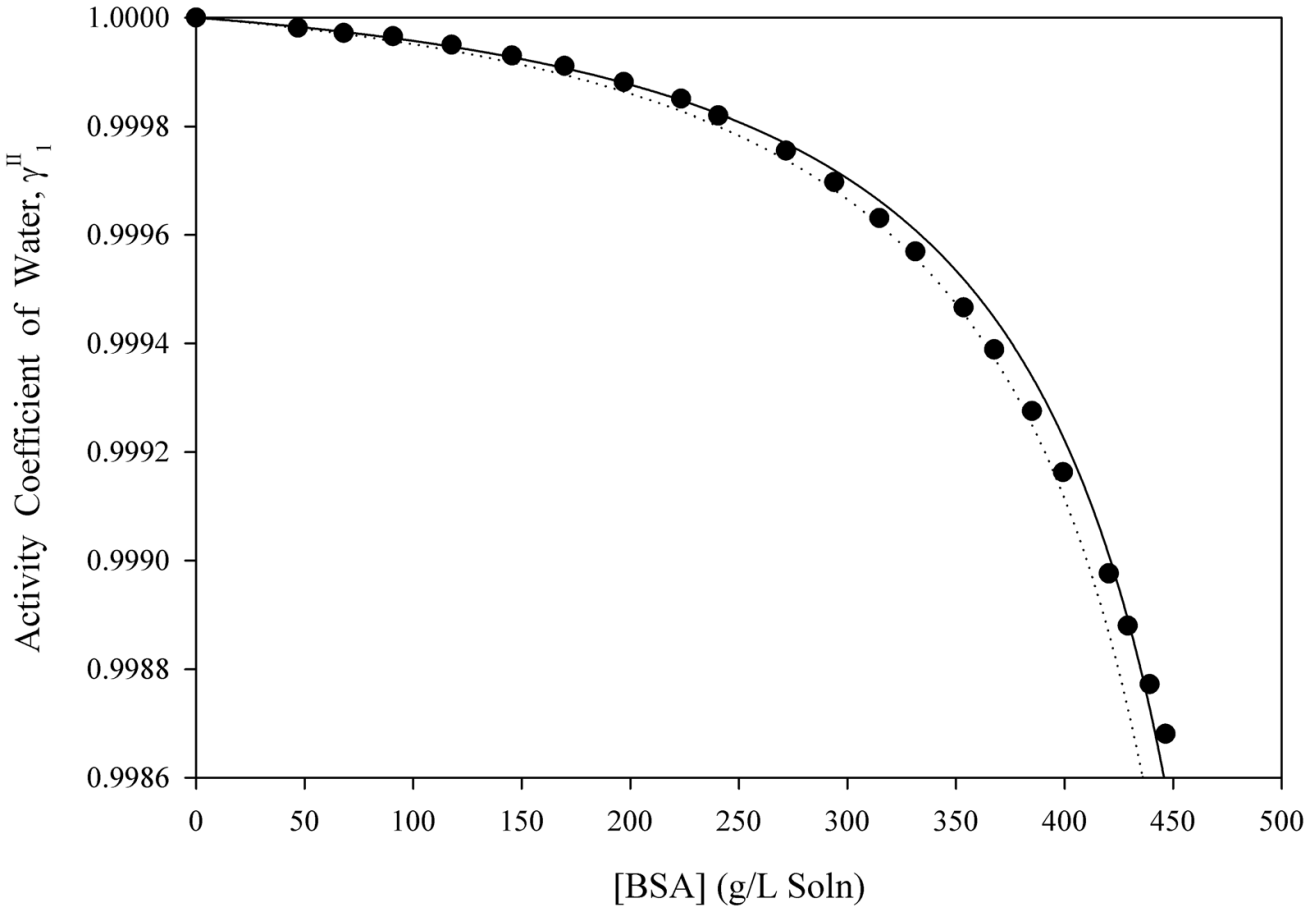
Activity Coefficient of Free-Solvent Using Literature Values of the Physical Parameters vs. Concentration for BSA in 0.15 M NaCl, pH 5.4. The calculated activity coefficients of BSA in 0.15(closed circles) are shown. The predicted activity coefficients (Eqn. 12) are plotted using the physical parameters available in literature for BSA in 0.15 M NaCl, pH 5.4 (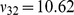 mol NaCl/mol BSA [6]): 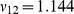 g H_2_O/g BSA (solid curve) and 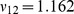 g H_2_O/g BSA (dotted curve).

**Figure 3:**
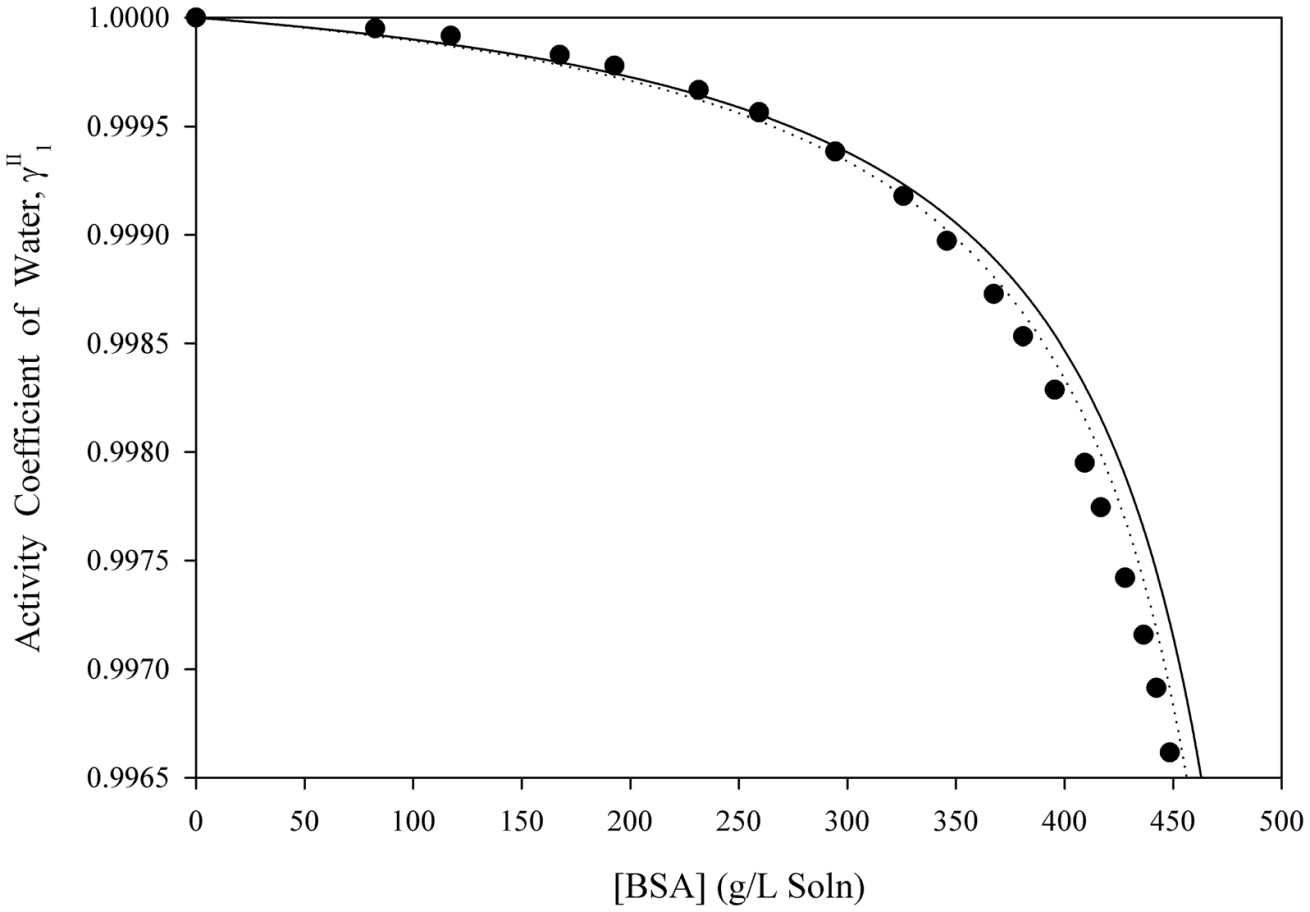
Activity Coefficient of Free-Solvent Using Literature Values of the Physical Parameters vs. Concentration for BSA in 0.15 M NaCl, pH 7.4. The calculated activity coefficients of BSA in 0.15(closed circles) are shown. The predicted activity coefficients (Eqn. 12) are plotted using the physical parameters available in literature for BSA in 0.15 M NaCl, pH 4.5 (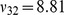 mol NaCl/mol BSA [6]): 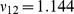 g H_2_O/g BSA (solid curve) and 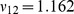 g H_2_O/g BSA (dotted curve).

**Figure 4:**
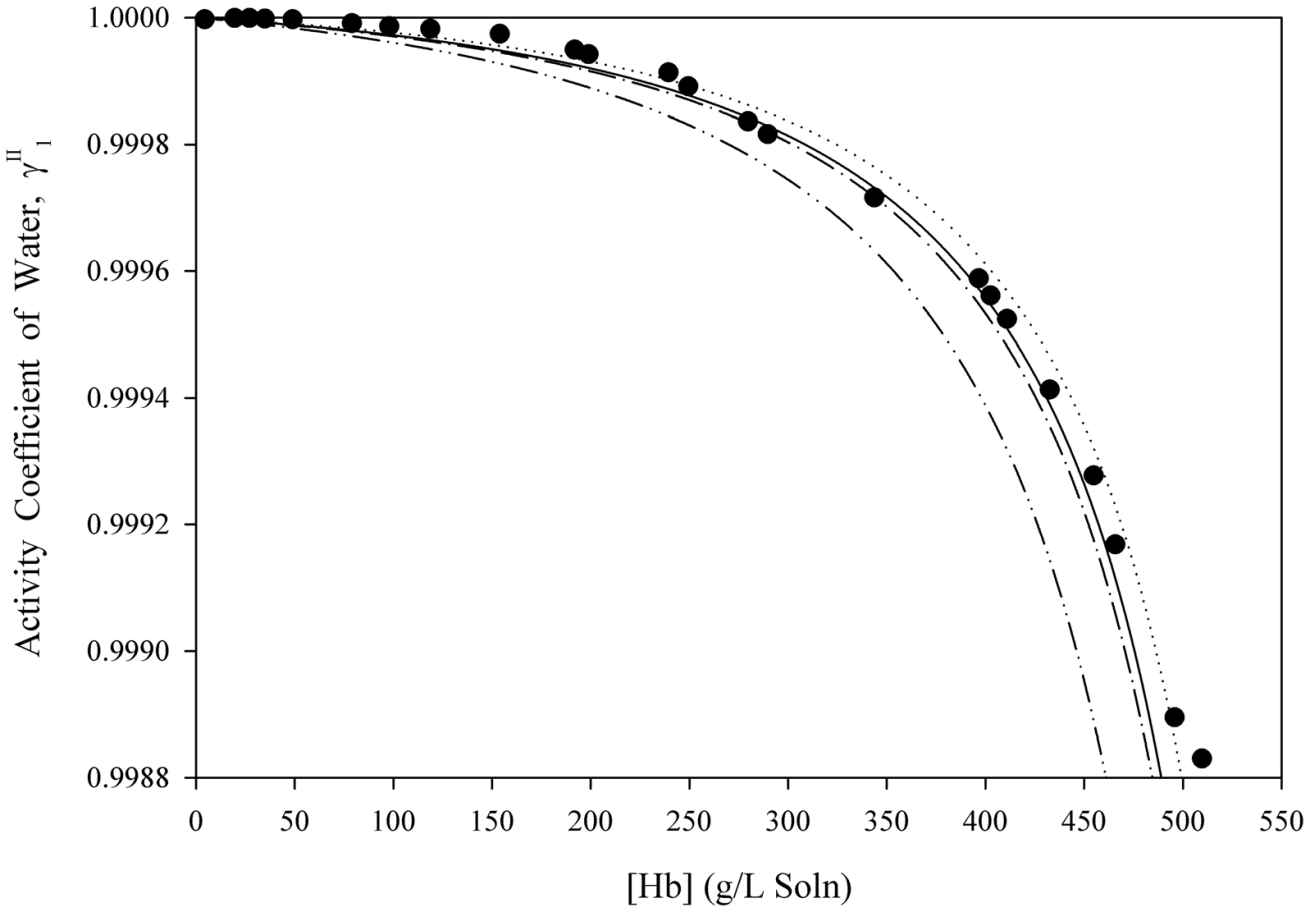
Activity Coefficient of Free-Solvent Using Literature Values of the Physical Parameters vs. Concentration for Hb. The calculated activity coefficients of Hb in 0.1(closed circles) are shown. The predicted activity coefficients (Eqn. 12) are plotted using the physical parameters available in literature for Hb (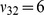 mol KCl/mol Hb [26]). The hydration values, determined from the SASA, are: 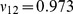 g H_2_O/g Hb (solid curve), 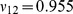 g H_2_O/g Hb (dotted curve), 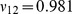 g H_2_O/g Hb (dash-dot curve), and 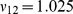 g H_2_O/g Hb (dash-dot-dot curve).

**Figure 5:**
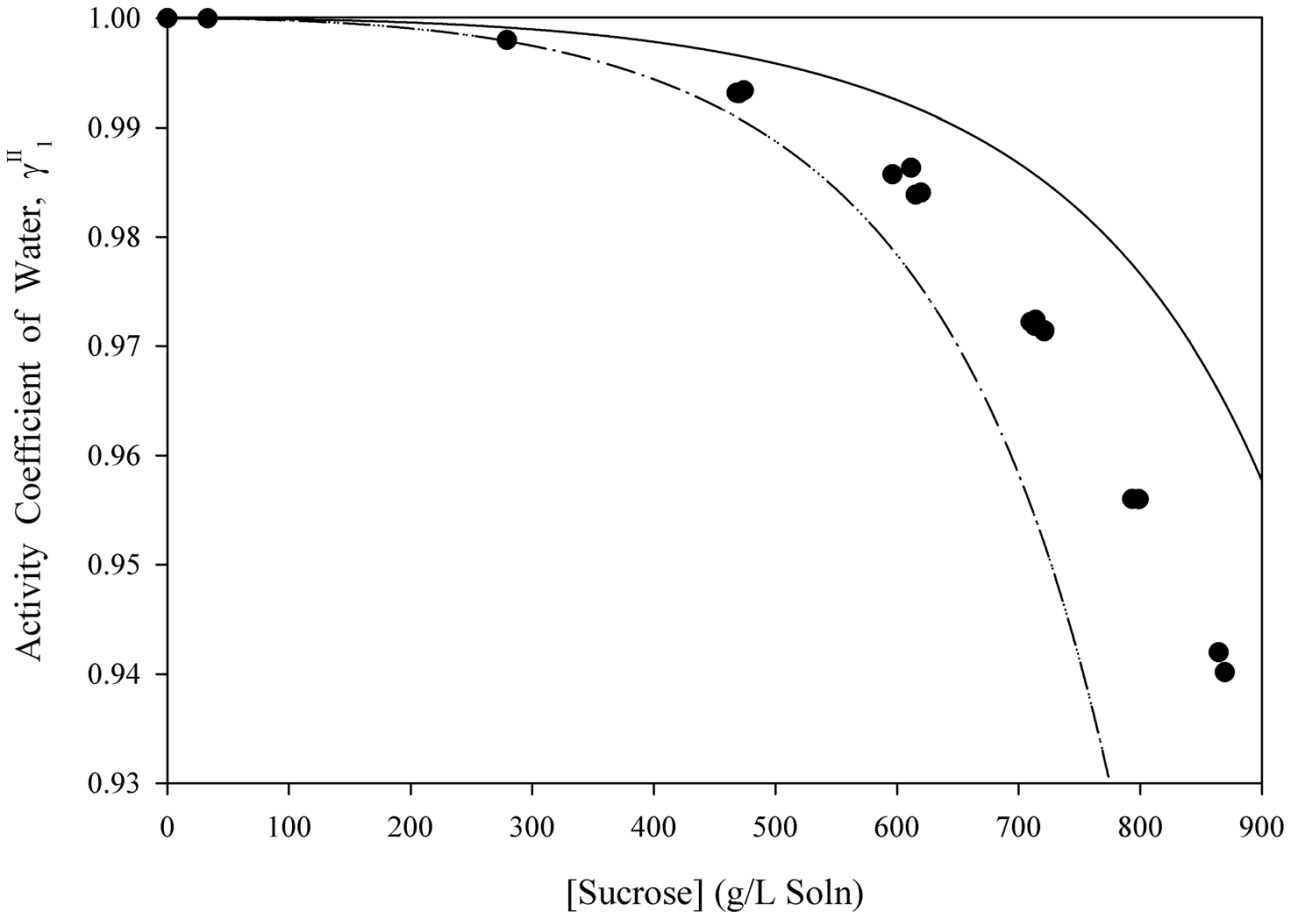
Activity Coefficient of Free-Solvent vs. Concentration for Sucrose. The calculated activity coefficients of sucrose in water (closed circles) are shown. The predicted activity coefficient (Eqn. 12) is plotted using the literature values of hydration (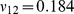 g H_2_O/g sucrose (solid curve) and 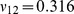 g H_2_O/g sucrose, (dotted curve)).

Eqn. 12 was used to estimate the activity coefficients of free-solvent for three separate macromolecules in aqueous solutions up to near-saturation concentrations. Figures 1, 2, 3, 4, 5 show the calculated activity coefficients of free-solvent (Eqn. 5), based on the osmotic pressure data, and the activity coefficients of free-solvent based on protein-solvent interactions (Eqn. 12) applied to three BSA solutions, one Hb solution, and one sucrose solution using only the physical parameters available in literature (Table 1).

**Table 1:** Physical parameters for BSA and Hb solutions.

Macromolecule (kDa)	Solution Properties Salt Conc., pH, Temp	Hydration, 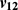 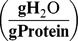	Ion Binding, 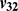 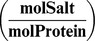
Sucrose (0.342)	H_2_O, 30°C	0.184-0.316 [10], [27], [28]	N/A
Bovine Serum Albumin (BSA) (66)	0.15 M NaCl, 4.5, 25°C	1.144 and 1.162 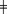	11.59 [6]
	0.15 M NaCl, 5.4, 25°C		10.62 [6]
	0.15 M NaCl, 7.4, 25°C		8.81 [6]
Sheep Hemoglobin (Hb) (69.7)	0.1 M KCl, 7.43, 0°C	0.973, 0.955, 0.981, and 1.025‡	6 (pH 7.4) [26]

^
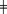
^ Based on the SASA from the homology model using GETAREA and MOLMOL, respectively.

‡ Based on the SASA from the available PDB using GETAREA, Swiss-Pdb Viewer (Quality 6), MOLMOL (Precision 6), and UCSF Chimera, respectively.

The hydration values are based on the SASA and assuming 15.2 molecules of water per nm^2^ surface area [9].

For all solutions, as the solute concentration increases, the activity coefficient of free-solvent decreases from unity as expected; the activity coefficient of free-solvent for a pure water solution should be unity. The calculated activity coefficients of free-solvent follow this trend for most of the solutions studied; however there is some deviation, which is most likely due to experimental error. The predicted activity coefficients of free-solvent based on protein-solvent interactions decrease from unity as the protein concentration increases for all five solutions studied.

The activity coefficients of free-solvent predicted based on protein-solvent interactions are compared to the calculated activity coefficients of free-solvent for BSA in 0.15 M NaCl, pH 4.5, 5.4, and 7.4 (Figures 1, 2, 3). The ion binding values are 11.59 mol NaCl/mol BSA, 10.62 mol NaCl/mol BSA, and 8.81 mol NaCl/mol BSA for BSA in 0.15 M NaCl, pH 4.5, 5.4, and 7.4, respectively [6]. The homology model SASA was computed using four molecular modeling software. The SASA are 28,065 Å^2^ (Swiss-Pdb Viewer [15]), 28,188 Å^2^ (MOLMOL [16]), 27,985 Å^2^ (VegaZZ [18]), and 27,746 Å^2^ (GETAREA [19]) and the corresponding hydration values are 1.157 g H_2_O/g BSA (Swiss-Pdb Viewer [15]), 1.162 g H_2_O/g BSA (MOLMOL [16]), 1.154 g H_2_O/g BSA (VegaZZ [18]), and 1.144 g H_2_O/g BSA (GETAREA [19]). The activity coefficient of free-solvent is predicted using the minimum and maximum hydration values (1.144 g H_2_O/g BSA and 1.162 g H_2_O/g BSA) and the corresponding ion binding values for each BSA solution. The predicted activity coefficients of free-solvent for all three BSA solutions follows the same trend as the calculated activity coefficients of free-solvent; the predicted activity coefficients are in excellent agreement with the calculated activity coefficients for BSA in 0.15 M NaCl, pH 5.4 and 7.4, and in good agreement for BSA in 0.15 M NaCl, pH 4.5.

The SASA from the molecular structure of Hb (PDB: 2QU0) for four of the molecular modeling software are 24,304 Å^2^ (Swiss-Pdb Viewer [15]), 24,981 Å^2^ (MOLMOL [16]), 26,100 Å^2^ (UCSF Chimera [17]), and 24,759 Å^2^ (GETAREA [19]) and the corresponding hydration values are 0.955 g H_2_O/g BSA (Swiss-Pdb Viewer [15]), 0.981 g H_2_O/g BSA (MOLMOL [16]), 1.025 g H_2_O/g BSA (UCSF Chimera [17]), and 0.973 g H_2_O/g BSA (GETAREA [19]). The activity coefficients of free-solvent were predicted for all four values of hydration using the literature value for ion binding, 6 mol KCl/mol Hb [26]. The predicted activity coefficients of free-solvent using the SASA from three of the molecular modeling software are in excellent agreement with the calculated activity coefficients, and the activity coefficients of free-solvent predicted using the SASA from UCSF Chimera [17] is in good agreement with the calculated values.

The predicted activity coefficients of free-solvent for sucrose are compared to the calculated activity coefficients of free-solvent from experimental osmotic pressure data (Figure 5). Many studies have determined the hydration of sucrose, with the most notable being those by Frazer and Myrick [10], Scatchard [27], and Einstein [28]. The range of sucrose hydration values is 5 – 6 mol H_2_O/mol sucrose [10], [27], [28]. The activity coefficients of free-solvent was predicted using the minimum and maximum values (within the range) of sucrose hydration: 0.184 g H_2_O/g sucrose (3.5 mol H_2_O/mol sucrose) and 0.316 g H_2_O/g sucrose (6 mol H_2_O/mol sucrose) [10], [27], [28].

## Discussion

The activity coefficient of free-solvent has now been given a physiological basis. Here, the activity coefficients of free-solvent are predicted for two macromolecules based on hydration and ion binding. As expected, the activity coefficients of free-solvent for all solutions decrease from unity as the protein concentration increases. Using the Gibbs-Duhem relationships, the activity coefficients of free-solvent can be used to determine the activity coefficients of the protein or the salt based on physically realistic parameters.

### Independently Determining the Physical Parameters of the Free-Solvent Model

The free-solvent model, which relies only on hydration and ion binding, has been shown to be remarkably robust due to the use of only physically realistic and independently measureable parameters.

The hydration of macromolecules has been extensively studied for several decades, including Einstein’s estimation of sucrose hydration from viscosity data [28]. Hydration can be determined using various methods, including ^17^O NMR [12], x-ray solution scattering [29], and small angle neutron scattering [29]. Furthermore, if structural information is known, the hydration value can be calculated assuming a monolayer of water surrounds the macromolecule [9].

The interaction between ions and proteins have also been examined using several techniques, electromotive force (EMF), distribution method, and isopiestic method, [24], [30]–[36] as well as mathematical models based on surface residues [24].

The developed model for the activity coefficient of free-solvent which relies on these two fundamental physical parameters is highly robust since, in addition to the independent methods for estimation of the values, protein hydration and protein-ion binding are also unique and do not rely on each other. Protein hydration is primarily dependent on the solvent accessible surface area, while protein-ion binding depends on surface residue charge, their location and neighboring residues, as well as the net charge of the protein.

### Crystallization Solution Properties of Bovine Serum Albumin

Herein, the BSA molecular structure, based on the homology model, was used for calculating the SASA due to the experimental conditions used for the crystallization of the molecular structures available in the Protein Data Bank for BSA (PDB: 3V03 and 4F5S). Both of the structures were crystallized at pH 6.5 in polyethylene glycol (PEG) solutions: 20% (w/v) PEG 3350 (PDB: 3V03) and 20 – 24% (w/v) PEG monomethyl ether (MME) 5000 (PDB: 4F5S). In addition, 200 mM Ca acetate and 100 mM Tris-HCl were used in the crystallization of BSA by Majorek *et al.* [20]; 150 – 300 mM NH_4_Cl and 100 mM 2-(N-morpholino)ehanesulfonic acid (MES) were used in the crystallization of BSA by Bujacz [21]. In the former case, the authors mention that monoclinic crystals were observed [20]; however, in the latter case, the authors state that the crystals were poor [21].

The effect of these solutions on the SASA compared to the SASA obtained from osmotic pressure for 0.15 M NaCl solutions is unknown. To investigate this, the osmotic pressure-based SASA can be determined for BSA in the crystallization solution properties.

Furthermore, the crystallization process, dehydrating the molecules, may have effects on the molecular structure due to charge repulsion. This is a very likely problem with the crystallization of BSA since it is a very negatively charged molecule in both of the crystallization solutions.

### Limitations of the Activity Coefficients of Free-Solvent Based on Protein-Solvent Interactions

Herein, the activity coefficient of free-solvent was only developed for protein solutions in which only solute-solvent interactions occur. However, for solutions in which protein-protein interactions occur, while Eqn. 12 is correct, the mole fraction of free-solvent in each compartment can be revised to include protein-protein interactions in order to determine the closed-form solution of the activity coefficients of free-solvent. This modification of the free-solvent model which accounts for protein-protein interactions in addition to the protein-solvent interactions, has been recently developed [37].

## Conclusion

A model for the activity coefficient of free-solvent was developed based on solute-solvent interactions. Unique about this approach is that this model uses no adjustable parameters and is based only on the independently determined physical parameters associated with protein hydration and ion binding. The closed-form solution for the single macromolecule, monovalent salt system activity coefficient of free-solvent is provided, and the predicted activity coefficient of free-solvent based on physical parameters from literature for three single macromolecule solutions, up to near-saturation concentrations, is shown.
